# Tsunami waves extensively resurfaced the shorelines of an early Martian ocean

**DOI:** 10.1038/srep25106

**Published:** 2016-05-19

**Authors:** J. Alexis P. Rodriguez, Alberto G. Fairén, Kenneth L. Tanaka, Mario Zarroca, Rogelio Linares, Thomas Platz, Goro Komatsu, Hideaki Miyamoto, Jeffrey S. Kargel, Jianguo Yan, Virginia Gulick, Kana Higuchi, Victor R. Baker, Natalie Glines

**Affiliations:** 1 Planetary Science Institute, 1700 East Fort Lowell Road, Suite 106, Tucson, AZ 85719-2395, USA; 2 NASA Ames Research Center, Mail Stop 239-20, Moffett Field, CA, 94035, USA; 3 Department of Planetology and Habitability, Centro de Astrobiología (CSIC-INTA), Madrid 28850, Spain; 4 Department of Astronomy, Cornell University, Ithaca, NY 14850, USA; 5 Astrogeology Science Center, U.S. Geological Survey, Flagstaff, AZ 86001, USA; 6 External Geodynamics and Hydrogeology Group, Department of Geology, Autonomous University of Barcelona, 08193 Bellaterra, Barcelona, Spain; 7 Planetary Sciences and Remote Sensing, Institute of Geological Sciences, Freie Universität Berlin, 12249 Berlin, Germany; 8 International Research School of Planetary Sciences, Università d’Annunzio, Viale Pindaro 42, 65127 Pescara, Italy; 9 The University Museum, University of Tokyo, 113-0033, Japan; 10 Department of Hydrology & Water Resources, University of Arizona, Tucson, AZ 85721, USA; 11 State Key Laboratory of Information Engineering in Surveying, Mapping and Remote Sensing, Wuhan University, Wuhan 430070, China; 12 SETI Institute, 189 Bernardo Avenue, Mountain View, CA 94043, USA

## Abstract

It has been proposed that ~3.4 billion years ago an ocean fed by enormous catastrophic floods covered most of the Martian northern lowlands. However, a persistent problem with this hypothesis is the lack of definitive paleoshoreline features. Here, based on geomorphic and thermal image mapping in the circum-Chryse and northwestern Arabia Terra regions of the northern plains, in combination with numerical analyses, we show evidence for two enormous tsunami events possibly triggered by bolide impacts, resulting in craters ~30 km in diameter and occurring perhaps a few million years apart. The tsunamis produced widespread littoral landforms, including run-up water-ice-rich and bouldery lobes, which extended tens to hundreds of kilometers over gently sloping plains and boundary cratered highlands, as well as backwash channels where wave retreat occurred on highland-boundary surfaces. The ice-rich lobes formed in association with the younger tsunami, showing that their emplacement took place following a transition into a colder global climatic regime that occurred after the older tsunami event. We conclude that, on early Mars, tsunamis played a major role in generating and resurfacing coastal terrains.

The existence of an early Mars northern ocean^1^,^2^,^3^,^4^,^5^,^6^,^7^ remains a fundamental mystery^8^,^9^. During the Hesperian Period (~3.71 to 3.37 Ga; ages herein based on Neukum chronology as given in Michael)^10^, Mars’ ancient hydrosphere was apparently cold-trapped within vast systems of subsurface aquifers underneath a thick, ice-rich permafrost zone^7^. Groundwater outbursts at the end of the Hesperian may have generated catastrophic floods that produced an ocean in the northern lowlands, as evidenced by a deposit that covers most of this region and generally exhibits a roughly topographically equipotential margin^1^,^2^,^3^,^4^,^5^,^6^,^7^,^11^,^12^. Radar-sounding data are consistent with the deposit being comprised of mostly water-ice^13^. This deposit is identified as the Late Hesperian lowland unit (*lHl*) on the latest geologic map of Mars^14^. However, until now, the lack of wave-cut paleoshoreline features^9^ and the presence of lobate margins^8^,^12^ appeared to be inconsistent with the Late Hesperian paleo-ocean hypothesis. Our new geologic mapping in Chryse Planitia and northwestern Arabia Terra regions reveals previously undistinguished, older and younger members of the unit (*lHl*_*1*_ and *lHl*_*2*_, respectively, Fig. 1A). Both members are bounded by south-facing lobes that are typically tens of kilometers in length and width; however, in Chryse Planitia these dimensions reach a few hundred kilometers in scale (Fig. 1B, Fig. S1). The lobes reach upland boundary surfaces distributed between approximately −4087 m and −3191 m of elevation (Fig. S2). These deposits embay dozens of streamlined promontories scattered over a surface area of ~570,000 km^2^ (Fig. 1B,C).

In THEMIS night-time infrared images, the upper reaches of the older deposit that were emplaced along Arabia and Tempe Terrae (member *lHl*_1_ (Fig. 1A)) appear thermally bright (i.e., rocky exposures)^15^ and abruptly transition upland-ward into thermally dark (i.e., fine-grained sediments)^15^ surfaces (e.g., Figs 2A,B and 3B). Close-up views show that the bright surfaces consist of boulder deposits, with individual boulders typically meters in diameter (Figs 2C and 3E, Fig. S3D). Exhumation of the boulder deposit from beneath ejecta blanket materials along impact crater rims (black arrows in Fig. 2B,C), as well as distinct onlapping contacts (e.g., Fig. S3C), show that the deposit overlies the thermally dark surfaces consisting of finer-grained materials (e.g., Figs 2A,B and 3B). Throughout spatially disconnected locations in the eastern part of northwestern Arabia Terra, the marginal parts of member *lHl*_1_ cover low-slope ramps that are extensively dissected by NNW-trending (Fig. S4A) sets of aligned channels (e.g., Fig. 3A–D). These channels were first identified in Viking data (but only locally along Arabia Terra in association with an older “lowland unit A”)^1^.

Upslope flows leading to the emplacement of the *lHl* unit are implied by the highland-facing orientation of the deposits’ lobes as well as their relief gains, which commonly are a few hundred meters (e.g., Fig. 1A,B, Figs. S5, S6). These characteristics rule out emplacement by gravity-driven downslope moving flows such as debris, flood, glacier and lava flows. Uphill unidirectional winds can generate elongate aeolian deposits known as wind streaks. However, these deposits are largely composed of saltating sand-sized lithic particles that are deposited in scattered patches on the lee sides of topographic obstacles (typically impact craters), exhibit surface bedforms, generally cover hills and mesas situated along their paths, and mostly have length-to-width ratios >1 (ref. 16). In contrast, the lobes of member *lHl*_*1*_ include boulders several meters in diameter (Figs 2C and 3E, Fig. S3D), and those of member *lHl*_*2*_ appear to be mostly composed of water-ice^6^,^12^,^13^,^14^. In addition, the lobes in both members diverge around numerous mesas (e.g., Fig. 1C) as well as broad rises (e.g., Fig. S3), and have length-to-width ratios mostly <1 (Fig. S1) (which is consistent with uphill flow along with substantial lateral spreading). Therefore, we propose that the two unit *lHl* members represent deposits emplaced by highly energetic, sediment-rich tsunami waves that originated from a Late Hesperian paleo-ocean.

In Deuteronilus Mensae, extensive troughs cut the boundary scarps covered by member *lHl*_*1*_. The troughs are locally intruded by member *lHl*_*2*_run-up lobes (e.g., Fig. 2D), indicating that they formed during the time interval separating the two tsunami events. Active resurfacing leading to the formation of these troughs likely lasted a few million years and could have been the result of Late Hesperian glacial erosion^17^. Crater-count statistics show that, while the deposits formed during the Late Hesperian Epoch, their absolute ages could differ as much as several tens of millions of years (see supplementary crater statistics).

The boulder deposits of member *lHl*_*1*_ drape over, and therefore postdate, the incision of adjoining highland channels (e.g., orange arrow in Fig. S3A), ruling out upland fluvial systems as possible discharge sources. Highly energetic, boulder-rich tsunami fronts on Earth show diversion around topographic obstacles as they propagate onshore^18^. Similarly, member *lHl*_*1*_ boulder deposits exhibit well-defined landward lobate margins around broad promontories (Fig. S3A–C). Member *lHl*_*1*_ boulders range from rounded to angular and are as much as ~10 m in diameter (Figs 2C and 3E, Fig. S3D), which are also characteristics of some terrestrial tsunami deposits^18^. Thus, we interpret the member *lHl*_*1*_lobes as made up of lowland and boundary clastic materials that were captured and transported by a tsunami wave, then beached farther inland as the wave lost its momentum.

Subsequently, we suggest that rapid gravity-forced backwash of the tsunami wave into the paleo-ocean dissected the channel systems on the marginal parts of member *lHl*_1_ in the eastern part of northwestern Arabia Terra (Fig. 3A–D). These channels have remarkable similarities to terrestrial tsunami backwash channels; including the presence of aligned channel heads^19^ (black arrows in Fig. 3C), perpendicular orientations to the reconstructed paleoshoreline^19^ (Fig. S4A), streamlined bars composed of reworked boulders^20^,^21^ (Fig. 3D,E), and widths ranging between ~50 and ~200 m (refs 19,22) (Fig. 3C,D). Parker *et al.*^23^ observed a few of these parallel channel systems in Arabia Terra using lower-resolution image data, and they also interpreted them as tsunami backwash channels.

The lower terminations of the proposed backwash channels are generally truncated by younger scarps (Figs 2D and 3C, Fig. S4A). However, the identification of a possibly subaqueously emplaced sedimentary lobe adjoining the lower reaches of a set of these channels located at ~−3795 m in elevation (Fig. S4B) provides an approximate upper boundary to the paleoshoreline from which the older tsunami propagated (Fig. 4A). The lowest margins of the mapped *lHl*_*2*_lobes are at ~−4100 m in elevation (Fig. S2), which we have used as an upper bound to the paleoshoreline elevation from which the younger tsunami propagated (Fig. 4B). The elevation difference between the two paleoshorelines implies a decrease in ocean level of ~300 m, which could have taken place via evaporation/sublimation within several million years^6^.

Based on these paleo-oceanographic reconstructions, we estimate that the areas inundated by the older and younger tsunamis within the study region were ~8 × 10^5^ km^2^ and ~1 × 10^6^ km^2^, respectively (Fig. S5). Measured typical run-up distances are tens to a few hundred kilometers for both the older and younger tsunamis, and their respective maxima reach ~529 km and ~650 km (Fig. S6). Overall, the morphometric characterizations of both tsunamis are strikingly similar. The slightly larger inundation area that was apparently covered during the younger event is consistent with the tsunami extending from a lower shoreline, and therefore, flowing over relatively smooth, older ocean and tsunami deposits. These run-up distances and inundation areas are enormous by terrestrial standards, which explain why the backwash channels exhibit lengths of ~20 km, while some terrestrial examples of backwash channel lengths produced by much smaller tsunamis range between ~200 and ~300 m in length^22^.

Our mapping (Fig. 1, Fig. S6) shows comparatively shorter run-up distances along the rougher and steeper cratered topography of the Arabia Terra boundary terrains, indicative of relatively lower wave heights and velocities, as predicted by tsunami numerical simulations^24^. These simulations also indicate that as the waves overflowed the Arabia Terra cratered boundary, their velocities would have abruptly dropped below the ~1 m/s threshold required to move multi-meter-scale boulders, explaining the occurrence of the boulder deposits in the region (Figs 2C and 3E, Fig. S3D). On the other hand, the more gentle slopes in Chryse Planitia would have resulted in a more gradual decrease in wave velocity, leading to the emplacement of more sorted sedimentary lobes, with their distal-most areas primarily consisting of finer-grained sediments. In addition, prior to their inundation by tsunami waves, the highland boundary surfaces were likely covered by extensive boulder fields, which would have been captured and redistributed by the waves, which is also another important factor accounting for the regional prevalence of boulder-rich lobes. In Chryse Planitia the tsunamis would have mostly propagated over gently-sloping plains that were largely made up of less bouldery outflow channel sedimentary deposits^14^.

The simulations also show that bolide impacts causing craters ~30 km in diameter would have generated tsunami waves with typical onshore heights of ~50 m and local variations from ~10 m to as much as ~120 m (ref. 24). Using run-up distances measured in 71 topographic profiles (Fig. S6), we have calculated the tsunami wave heights and find that they reasonably match the simulations’ predicted ranges^24^ (see supplementary calculations). In addition, whereas the simulations do not describe the hydrodynamic behavior of the backwash stage, the formation of several marine impact craters on Earth has also resulted in documented tsunami backwash channels^25^.

Using the surface area of the paleo-ocean’s region included in the numerical simulation by Iijima *et al.*^24^ (i.e., ~4.5 × 10^6^ km^2^) and the crater production function of Ivanov^26^, we find that ~23 marine impact craters ≥30 km in diameter would have formed within this part of Mars during the Late Hesperian Epoch (3.61–3.37 Ga)^10^,^27^. Of these, 7 fit in the diameter range of 30–35 km, which was used in the tsunami simulations^24^. The prediction is that, within the particular region of the ocean analyzed here, on average about 2 impact craters ~30 km in diameter formed every 30 million years during this time period. Therefore, within statistical constraints for the deposits’ surface ages and for crater production rates, impacts can account for generation of both *lHl* members as tsunami deposits (see supplementary crater statistics).

Briny aqueous chemistry models show that the ocean could have remained in liquid form over millions of years, and consequently mostly free of an ice cover even during cryogenic climatic conditions^28^. Another geologic scenario invokes the formation of an ice-covered ocean soon after the ocean’s emplacement^6^. However, no numerical simulations have been performed to detail the behavior of impact-related tsunamis^24^ on these types of Martian marine environments.

High rates of marine, and subsequent periglacial^6^,^12^,^14^ resurfacing, likely reduced the topography of the tsunami-generating crater structures. Such resurfacing can also explain the lack of well-preserved impact craters predating the Amazonian Period in the northern lowlands^12^. The frequency rate of ~30 km in diameter impact craters for the entire ocean’s surface area (~24 × 10^6^ km^2^, as determined by Head *et al.*^3^) is one every 2.7 million years during the Late Hesperian. Although we have only identified evidence for two tsunami events in our study area, other regions in the northern plains likely experienced similar tsunami-related coastal resurfacing, perhaps associated with other impacts, huge landslides, or large marsquakes. Older but less extensive tsunami deposits may have been completely resurfaced by more recent events with similar run-up distances. Thus, the mapped tsunami margins comprise only the largest magnitude tsunami events located at the highest elevations.

Many of the *lHl*_*1*_ lobes are mostly made up of lithic deposits and exhibit backwash modifications. In contrast, the landward-facing lobate termini of unit *lHl*_*2*_ lack evidence indicative of a backwash phase subsequent to their emplacement. Like on Earth, the absence of backwash features associated with these flows could have been the result of the waves transitioning into sub-aerial sediment-laden slurry flows extending over low gradient surfaces^29^,^30^ (supplementary calculations), which can also explain the presence of possible contractional folds along the margins of some of the member’s lobes (e.g., black arrow in Fig. 2D). However, the *lHl*_*2*_lobes appear to be mostly composed of water-ice^6^,^12^,^13^,^14^, suggesting that the transition into slurry likely involved the formation and incorporation of a significant proportion of ice particles. In May 2013, the Saskatchewan Water Security Agency filmed an ice surge in the Codette Reservoir near Nipawin, Saskatchewan, Canada. The surge comprises a spectacular terrestrial analog of rarely observed catastrophic ice-rich flows leading to the emplacement of enormous lobate fronts, which are remarkably similar to those of member *lHl*_*2*_(video link included in ref. 31).We propose that these morphologic differences might be linked to colder environmental conditions following the first tsunami event.

Our mapping of two unit *lHl* members as tsunami lobes is consistent with the occurrence of two paleoshoreline levels of a receding Martian northern plains ocean during the Late Hesperian (Fig. 4, Fig. S5). However, resurfacing by the tsunami waves has obscured the paleoshorelines, thus making rigorous testing of their equipotentiality impossible.

## Mapping Methodology

**Figure 2:**
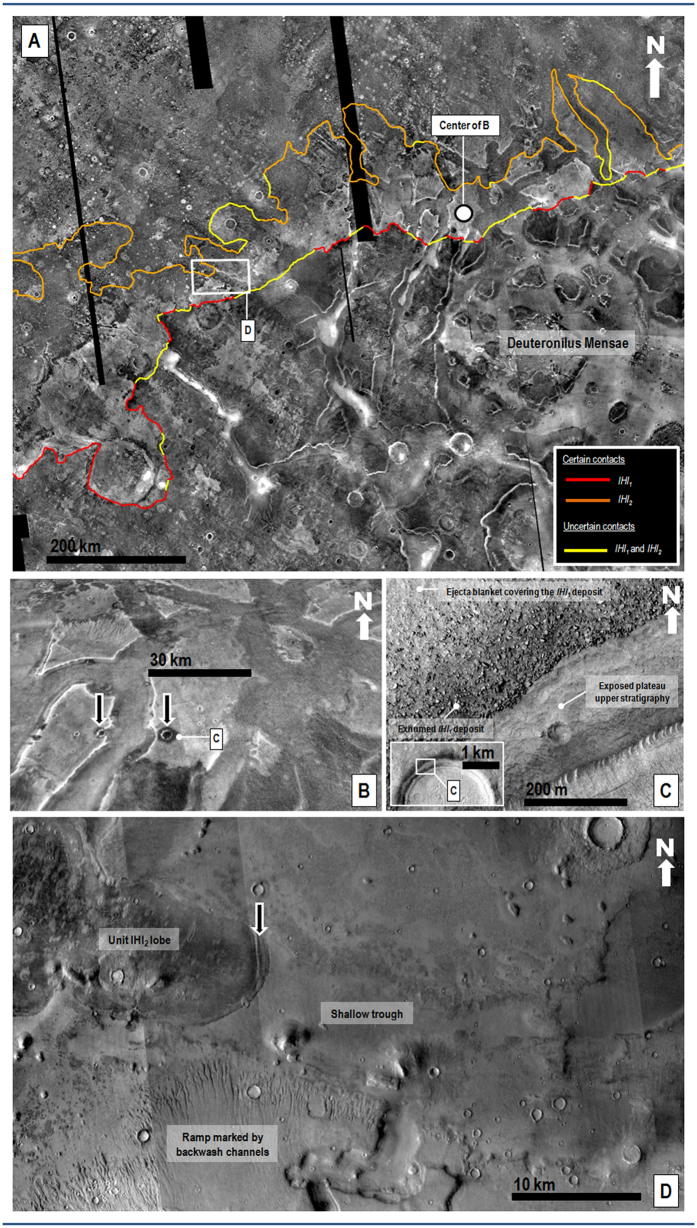
**(A)**View of highland surfaces along the highland-lowland boundary in northwestern Arabia Terra, which are embayed by the upper reaches of members *lHl*_1_ and *lHl*_2_. The red (*lHl*_1_) and orange (*lHl*_2_) lines show contact sections mapped as certain. The yellow lines are zones where the contacts are obscured due to localized resurfacing or mantling. Out of the total 4124 km length of these mapped contacts, 76% is certain and 23% is uncertain. Part of THEMIS night-time infrared image mosaic (100 m/pixel) centered at 43°26′N, 12°29′E. Credit: Christensen *et al.*^32^. **(B)** Perspective close-up view on a system of mesas, which are partly covered by the upper reaches of member *lHl*_1._ These deposits consist of thermally bright materials that pass upland-wards into thermally dark surfaces. The black arrows show locations where the thermally dark materials are exhumed from beneath the thermally bright materials by impact craters. Center location indicated by dot in panel A. Part of THEMIS night-time infrared mosaic (100 m/pixel) centered at 45°45′N, 16°41′E. Credit: Christensen *et al.*^32^. **(C)** Visible light close-up view on one of the craters identified in panel (**B)**. The impact crater’s inner wall shows the boulder deposit (member *lHl*_1_) overlying an exhumed non-bouldery upper stratigraphy. The boulder deposit is also locally covered by a non-bouldery ejecta blanket. The inset shows the view’s location along the crater’s northern margin. Part of HiRISE image ESP_017355_2260 centered at 45.82°N 16.47°E (50 cm/pixel). Credit: NASA/JPL/University of Arizona. (**D**) Close-up view of northeast Deuteronilus Mensae showing a low-angle ramp marked by backwash channels (part of member *lHl*_1_), which is cut by a shallow trough. A lobe of member *lHl*_2_ can be observed to run up the trough’s surface. The black arrow identifies a marginal compressional fold. Location is shown in panel **(A)**. Part of a CTX mosaic centered at 44°49′N, 11°10′E. Credit: NASA/JPL. The license terms can be found on the following link: pds-imaging.jpl.nasa.gov/portal/mro_mission.html. We produced the mosaics and maps in this figure using Esri’s ArcGIS^®^ 10.3 software (http://www.esri.com/software/arcgis).

Mapping in this investigation was performed using Esri’s ArcGIS^®^ 10.3 software (http://www.esri.com/software/arcgis). Embayment and overlapping relationships leading to the recognition of the outer margins of members *lHl*_1_ and *lHl*_2_ involved an integrated analysis of (1) thermal infrared image data (i.e., Mars Odyssey Thermal Emission Imaging System (THEMIS) night-time and day-time infrared image mosaics (100 m per pixel)), (2) visible image data (i.e., Mars Reconnaissance Orbiter Context Camera (CTX, (5.15–5.91 m/pixel)) images, and (3) Mars Global Surveyor Mars Orbital Laser Altimeter (MOLA, ~460 m/pixel horizontal and ~1 m vertical resolution) digital elevation models. In some areas, contacts are buried underneath ejecta blanket materials or are locally resurfaced; we mapped these sections as uncertain contacts (Fig. 2A).

## Additional Information

**How to cite this article**: Rodriguez, J. A. P. *et al.* Tsunami waves extensively resurfaced the shorelines of an early Martian ocean. *Sci. Rep.* **6**, 25106; doi: 10.1038/srep25106 (2016).

## Supplementary Material

Supplementary Figures

Supplementary Calculations

Supplementary Crater Statistics

## Figures and Tables

**Figure 1:**
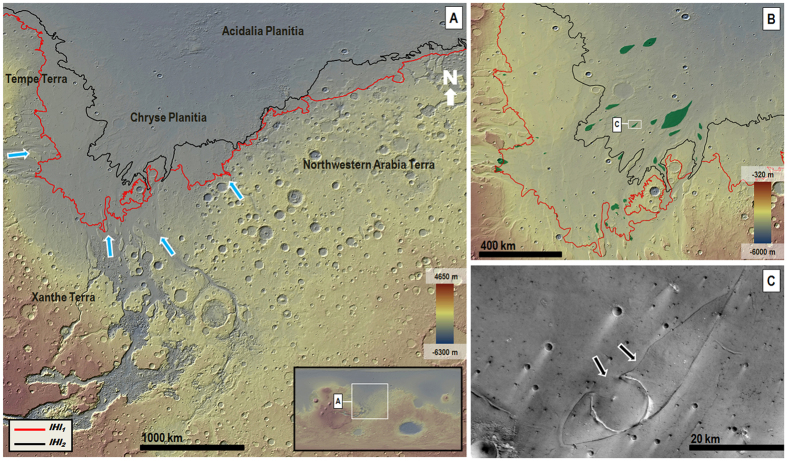
(**A**) View of the circum-Chryse highland-lowland boundary region, which is made up of the Chryse and Acidalia Planitiae lowlands and the Tempe, Xanthe, and Arabia Terrae highlands (inset shows region within the planetwide context). The boundary is breached by the planet’s largest outflow channels (blue arrows). The red and black lines trace the margins of Late Hesperian lowland members *lHl*_1_ and *lHl*_2_, respectively. **(B)** View of Chryse and Acidalia Planitiae showing the distribution of streamlined promontories (green) buried by these deposits. The base images for panels (**A**,**B)** are color-coded shaded-relief MOLA digital elevation models (460 m/pixel). Credit: MOLA Science Team, MSS, JPL, NASA. **(C)** View of a streamlined promontory that is embayed, and partly buried (e.g., black arrows), by member *lHl*_2_ materials. Part of HRSC image H1436_0000_ND3 (12.5 m/pixel) centered at 30.3°N, 35.9°W, (http://hrscview.fu-berlin.de/cgi-bin/ion-p?page=product.ion&mage=1436_0000), which is licensed under Attribution-ShareAlike 3.0 IGO license. The license terms can be found on the following link: https://creativecommons.org/licenses/by-sa/3.0/igo/. Credit: ESA/DLR/FU Berlin. We produced the mosaics and maps in this figure using Esri’s ArcGIS^®^ 10.3 software (http://www.esri.com/software/arcgis).

**Figure 3:**
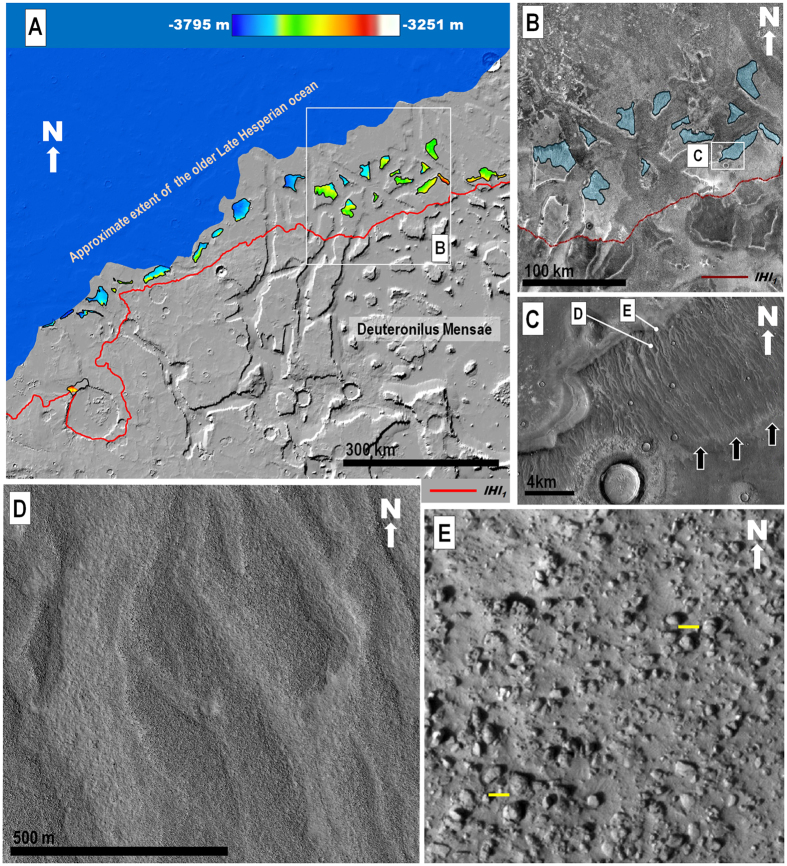
**(A)** View of Deuteronilus Mensae in northwestern Arabia Terra showing the distribution and elevation ranges of ramp surfaces marked by backwash channels as well as the reconstructed location of the paleoshoreline from which the older tsunami (member *lHl*_*1*_) propagated (see Fig. 4 and Fig. S4 for details on paleoshoreline reconstructions). Shaded-relief MOLA digital elevation model (460 m/pixel). Credit: MOLA Science Team, MSS, JPL, NASA. **(B)** Close-up view on panel (**A**) showing the distribution of some of the channel-scoured, north-sloping highland mesas (blue areas). Channels occur on bright (i.e., higher thermal inertia) surfaces, which abruptly transition (red line) in lower part of image to uplands covered by dark (lower thermal inertia) materials. THEMIS night-time infrared image mosaic, context and location in panel **A**. Credit: Christensen *et al.*^32^. Example of channeled surface (**C**) displaying streamlined bars (**D**) made up of rounded to angular boulders as much as ~10 m in diameter (yellow bars are 10 m in length) (**E**). (**C–E)**, Parts of HiRISE image ESP_028537_2270, 25 cm/pixel. Credit: NASA/JPL/University of Arizona.) We produced the mosaics and maps in this figure using Esri’s ArcGIS^®^ 10.3 software (http://www.esri.com/software/arcgis).

**Figure 4:**
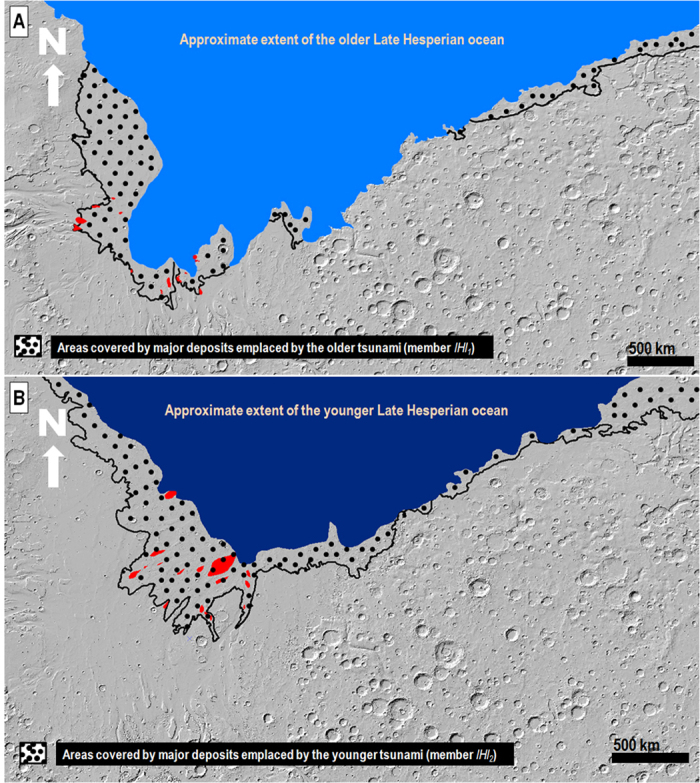
**(A)** Late Hesperian ocean with a paleoshoreline elevation close to −3795 m (light blue), and estimated extent of the older tsunami (dotted surface). **(B)** Late Hesperian ocean with a paleoshoreline elevation close to −4100 m (dark blue), and estimated extent of the younger tsunami (dotted surface). The ocean margins were reconstructed by tracing the estimated paleoshoreline elevations, while carefully interpolating across topography generated by clearly younger resurfacing processes. The tsunami margins were produced using the *lHl* unit’s lobes extending from the projected paleoshoreline elevations. Areas marked in red represent the locations of streamlined promontories in Chryse Planitia, which were embayed, and/or buried, by materials emplaced during each of the tsunami events. Topography in (**A**,**B)** from shaded-relief MOLA digital elevation model (460 m/pixel). Credit: MOLA Science Team, MSS, JPL, NASA. We produced the mosaics and maps in this figure using Esri’s ArcGIS^®^ 10.3 software (http://www.esri.com/software/arcgis).
