# Supplementary material for: Tsunami waves extensively resurfaced the shorelines of an early Martian ocean
Source: Sci Rep. 2016 May 19;6:25106. doi: 10.1038/srep25106 (PMC4872529; doi:10.1038/srep25106)
Supplement: Supplementary Figures [file srep25106-s1.doc]

**Supplementary figures to: Tsunami waves extensively resurfaced the shorelines of an early Martian ocean**

J. Alexis P. Rodriguez^1,2^, Alberto G. Fairén^3,4^, Kenneth L. Tanaka^5^, Mario Zarroca^6^, Rogelio Linares^6^, Thomas Platz^1,7^, Goro Komatsu^8^, Hideaki Miyamoto^9^, Jeffrey S. Kargel^10^, Jianguo Yan^11^, Virginia Gulick^2,12^, Kana Higuchi^3^, Victor R. Baker^10^, Natalie Glines^2,12^

*^1^Planetary Science Institute, 1700 East Fort Lowell Road, Suite 106, Tucson, AZ 85719-2395, USA.*

*^2^NASA Ames Research Center, Mail Stop 239-20, Moffett Field, CA, 94035, USA.*

*^3^Department of Planetology and Habitability, Centro de Astrobiología (CSIC-INTA), Madrid 28850, Spain.*

*^4^Department of Astronomy, Cornell University, Ithaca, NY 14850, USA.*

*^5^Astrogeology Science Center, U.S. Geological Survey, Flagstaff, AZ 86001, USA.*

*^6^External Geodynamics and Hydrogeology Group, Department of Geology, Autonomous University of Barcelona , 08193 Bellaterra, Barcelona, Spain.*

*^7^Planetary Sciences and Remote Sensing, Institute of Geological Sciences, Freie Universität Berlin, 12249 Berlin, Germany.*

*^8^International Research School of Planetary Sciences, Università d’Annunzio, Viale Pindaro 42, 65127 Pescara, Italy.*

*^9^The University Museum, University of Tokyo, 113-0033, Japan.*

*^10^Department of Hydrology & Water Resources, University of Arizona, Tucson, AZ 85721, USA.*

*^11^State Key Laboratory of Information Engineering in Surveying, Mapping and Remote Sensing, Wuhan University, Wuhan 430070, China.*

*^12^SETI Institute, 189 Bernardo Avenue, Mountain View, CA 94043, USA.*


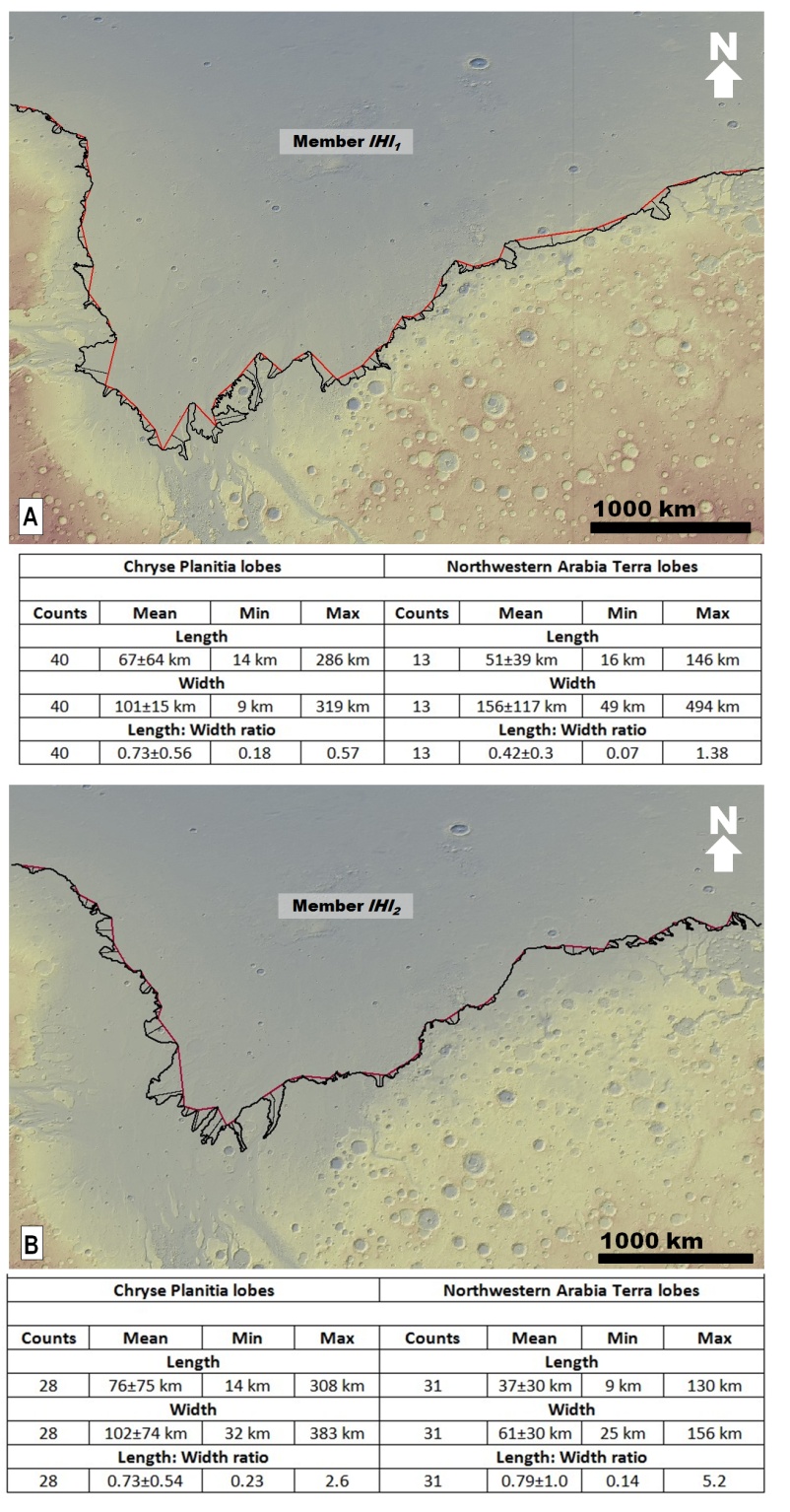


**Fig. S1** Characterization of the length-width ratios for the lobes in unit members *lHl*_1_ **(A)** and *lHl*_2_ **(B)**. The lobe bases were traced by connecting the local maxima of concave down zones. The length of each lobe was measured by determining the axial distance between their bases and the most distal concave up terminal upper reaches. High value statistical outliers correspond to lobes emplaced within channels. Note that ± values are 1-σ standard deviations. Panels A and B are Digital Elevation Models from color-coded MOLA topography (460 m/pixel). Credit: MOLA Science Team, MSS, JPL, NASA. We produced the mosaics, maps and measurements in this figure using [Esri](http://en.wikipedia.org/wiki/Esri)'s ArcGIS® 10.3 software (<http://www.esri.com/software/arcgis>).


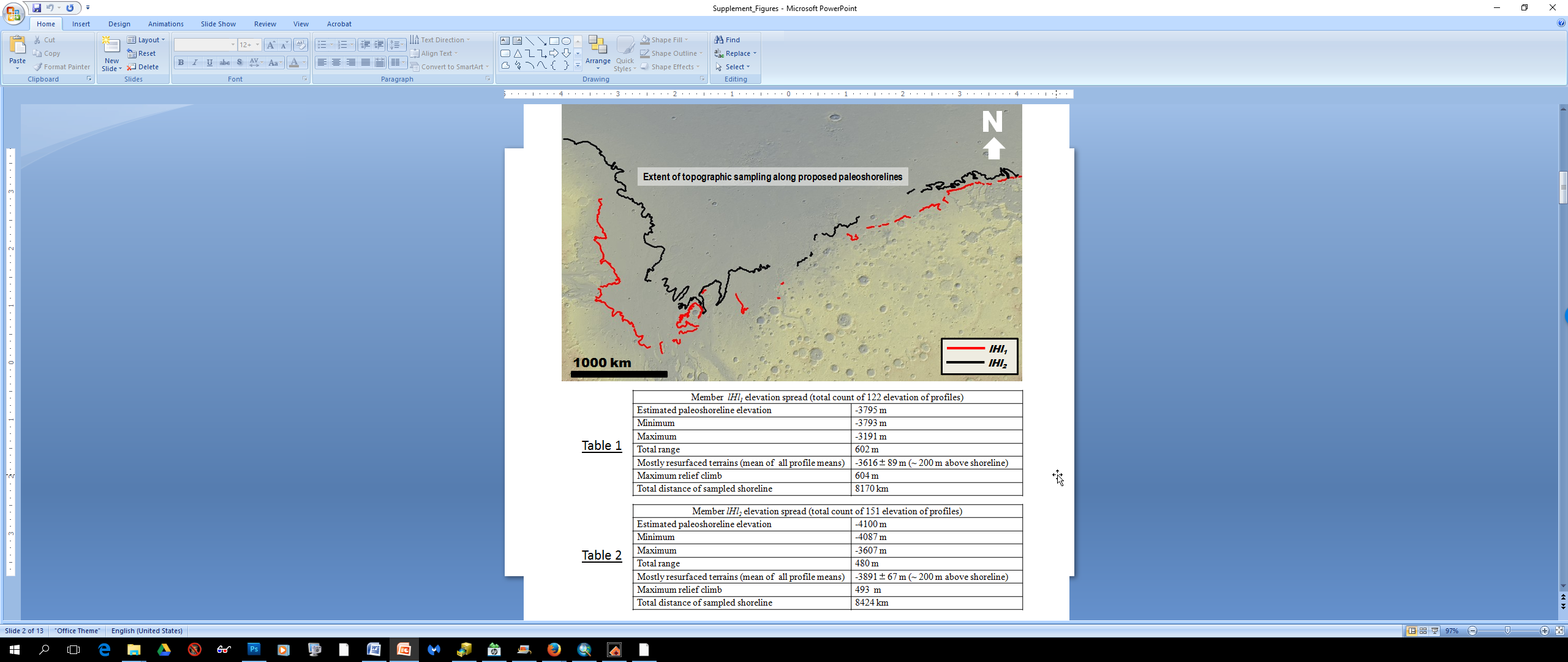
**Fig. S2** Map showing sections of the mapped contacts for members *lH1_1_* and *lHl_2_*, which do not exhibit significant topographic modifications by processes such as impact cratering, erosion by valleys, and collapse. These selected contacts were subsequently converted to elevation profiles, which were then exported as tables for statistical analysis. Note that ± values are 1-σ standard deviations. Digital Elevation Model from color-coded MOLA topography (460 m/pixel). Credit: MOLA Science Team, MSS, JPL, NASA. We produced the mosaics, maps and measurements in this figure using [Esri](http://en.wikipedia.org/wiki/Esri)'s ArcGIS® 10.3 software (<http://www.esri.com/software/arcgis>).


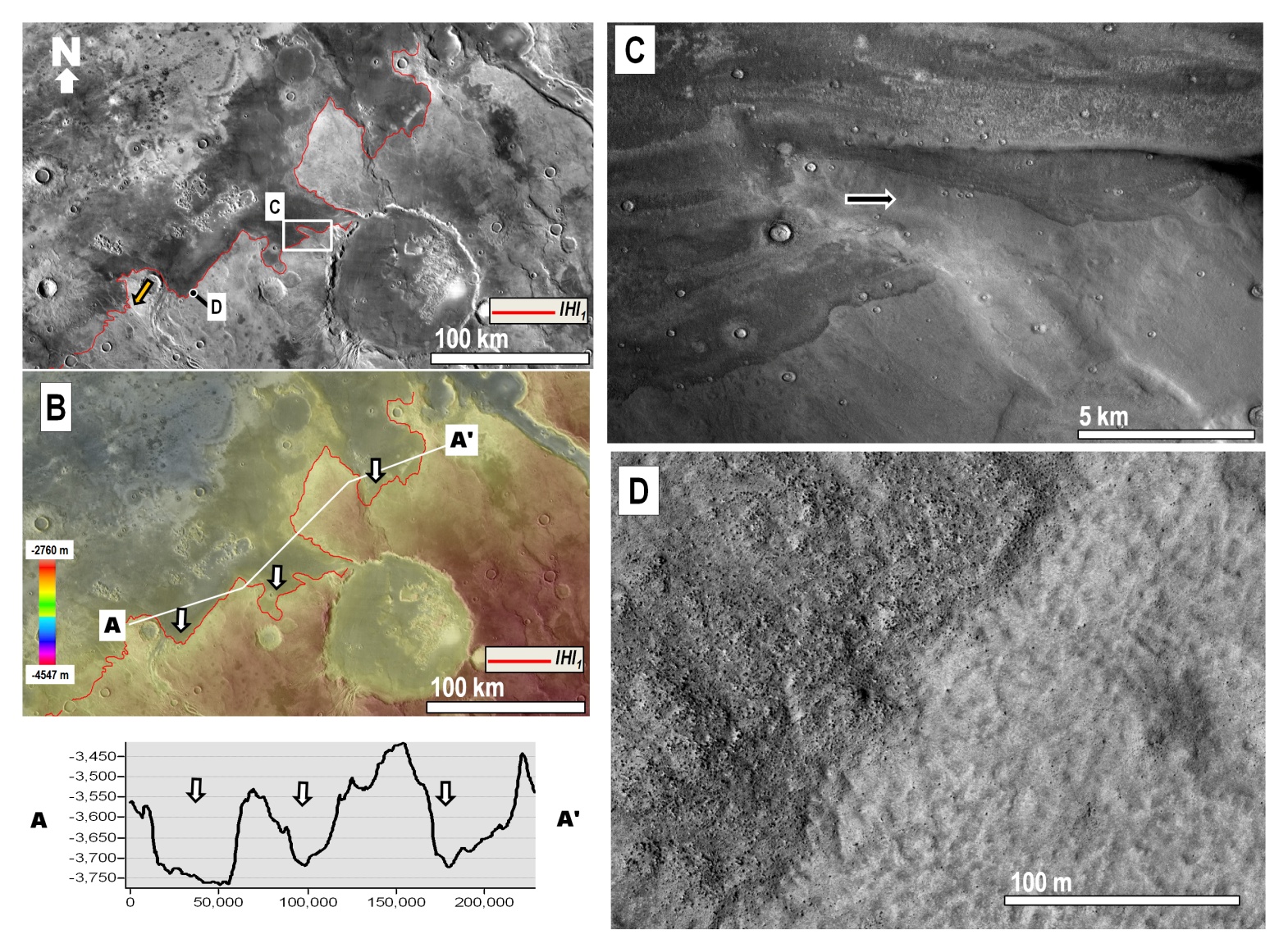
**Fig. S3 (A)** Boundary region in northwestern Arabia Terra centered at 42° 0' N, 6° 17' E. The red line traces the upper boundary of member *lHl_1_*, which in this region forms an extensive boulder deposit. The orange arrow identifies the lower reaches of channels, which are covered by the deposit’s margin. Part of regional THEMIS day-time infrared image mosaic (100 m/pixel). Credit: Christensen et al.^1^. **(B)** Topographic drape over image in panel A shows that the deposit’s lobes were produced by flows into upland embayments (white arrows in image and elevation profile). Both axes in the elevation profile are in meters. MOLA DEM (460 m/pixel). Credit: MOLA Science Team, MSS, JPL, NASA. **(C)** Close-up view showing evidence for upslope flow separation around a promontory (black arrow) significantly smaller than those shown in panel A. Part of a regional CTX mosaic. Credit: NASA/JPL. The license terms can be found on the following link: pds-imaging.jpl.nasa.gov/portal/mro_mission.html. **(D)** View of the deposit’s margin over the adjoining upland surface showing multi-meter scale boulders. HiRISE image PSP_006464_2220, 25 cm/pixel. Credit: NASA/JPL/University of Arizona. We produced the mosaics, maps and measurements in this figure using [Esri](http://en.wikipedia.org/wiki/Esri)'s ArcGIS® 10.3 software (<http://www.esri.com/software/arcgis>).

**
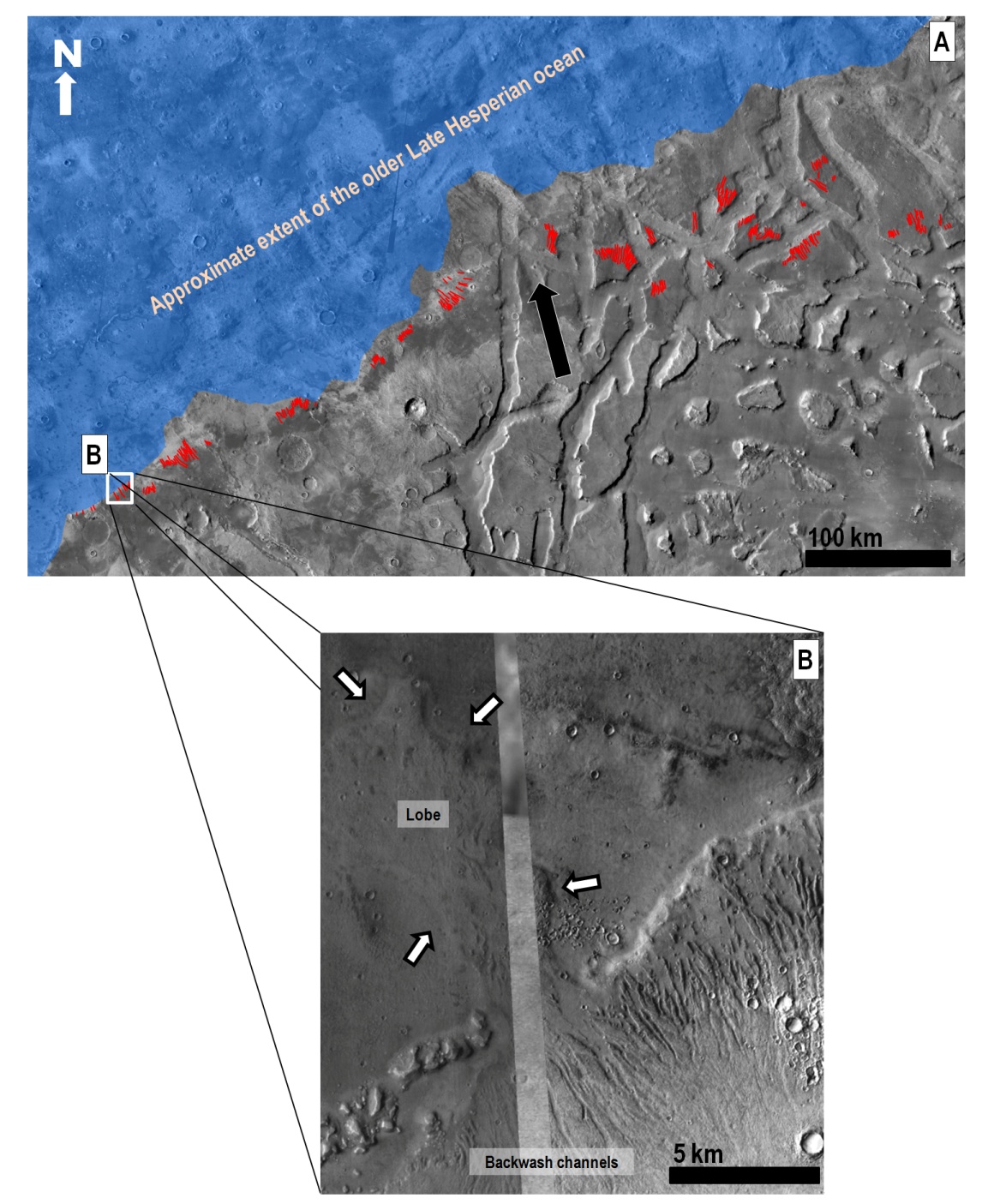
**

**Fig. S4 (A)** Linear directional mean (black arrow) generated from a total of 315 backwash channels in northwestern Arabia Terra (red lines). Part of regional THEMIS day-time infrared image mosaic (100 m/pixel) centered at 45°7'N, 14°38'E. Credit: Christensen et al.^1^. We produced the mosaic, map and linear directional mean calculation in this panel using [Esri](http://en.wikipedia.org/wiki/Esri)'s ArcGIS® 10.3 software (<http://www.esri.com/software/arcgis>). **(B)** Close-up view on the lower reaches of a set of these channels, which at approximately -3795 m, transition into a depositional lobe (white arrows). The lobe’s surface locally retains the termini of some of the backwash channels, indicating that its emplacement could have been related to the backwash flows losing their momentum upon entering the ocean’s interface. Thus, the elevation of this transitional contact likely represents that of the paleo-ocean’s margin. Part of a CTX mosaic centered at 43°43'N, 7°49'E. Credit: NASA/JPL. The license terms can be found on the following link: pds-imaging.jpl.nasa.gov/portal/mro_mission.html.

**
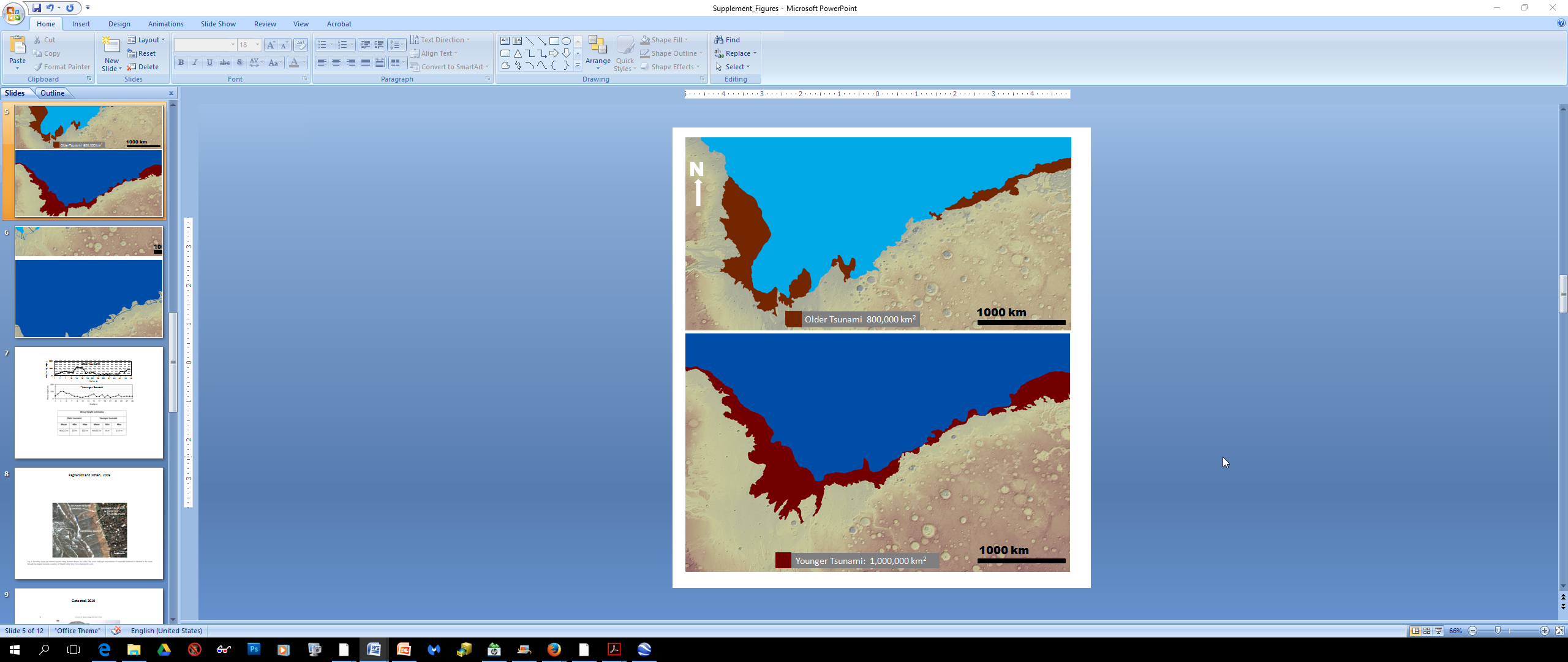
**

**Fig. S5** Inundated surfaces for both tsunami events. Digital Elevation Models from color-coded MOLA topography (460 m/pixel). Credit: MOLA Science Team, MSS, JPL, NASA. We produced the mosaics, maps and measurements in this figure using [Esri](http://en.wikipedia.org/wiki/Esri)'s ArcGIS® 10.3 software (<http://www.esri.com/software/arcgis>).


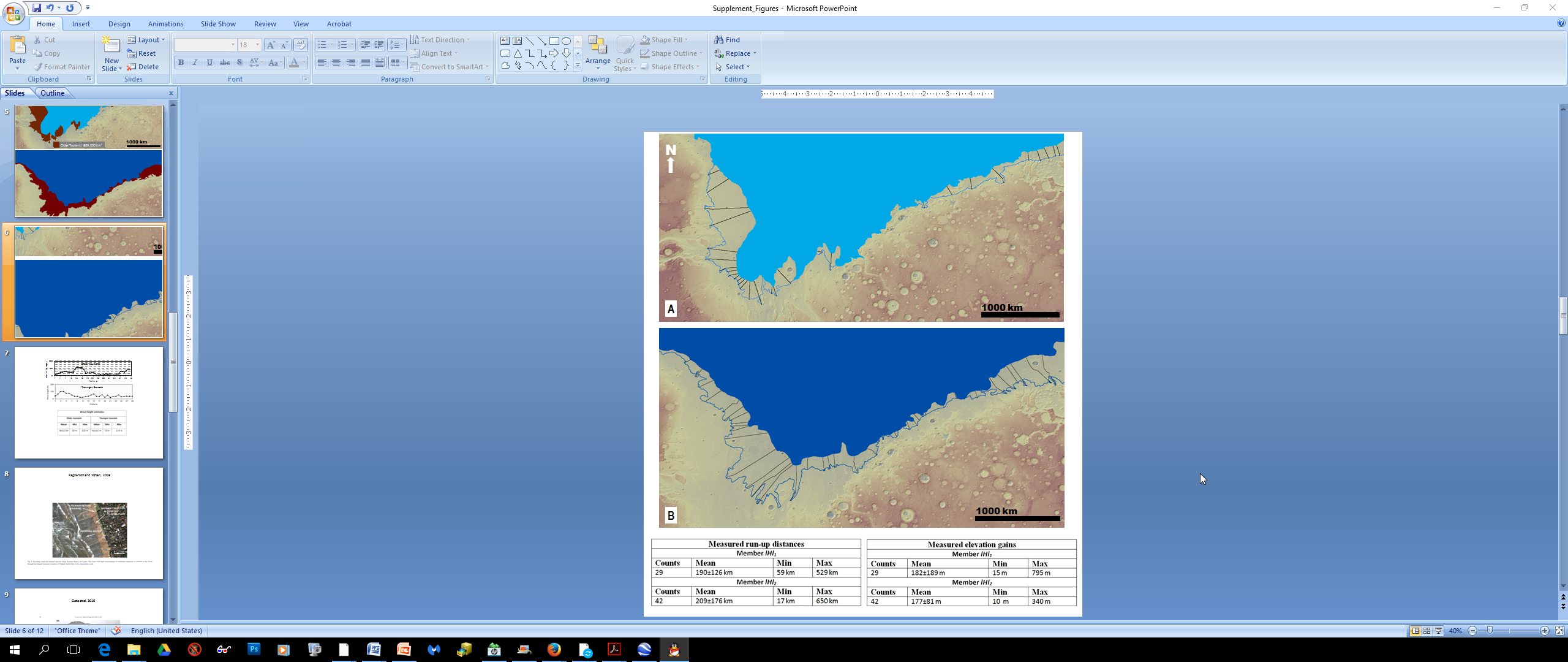
**Fig. S6** Characterization of tsunami run-up distances (i.e., Xmax in numerical supplement) and elevation gains using elevation profiles extending perpendicular to the proposed older (A) and younger (B) paleoshorelines. The statistical output shows typical run-up distances and elevation gains of ~200 km and ~180 m, respectively, but with significant regional variability. Note that ± values are 1-σ standard deviations. Digital Elevation Models from color-coded MOLA topography (460 m/pixel). Credit: MOLA Science Team, MSS, JPL, NASA. We produced the mosaics, maps and measurements in this figure using [Esri](http://en.wikipedia.org/wiki/Esri)'s ArcGIS® 10.3 software (<http://www.esri.com/software/arcgis>).

**References**

1 Christensen, P.R. *et al.* THEMIS Public Data Releases, Image Explorer, (2006), (Date of access: 11/24/2015), Planetary Data System node, Arizona State University, [http://themis-data.asu.edu](http://themis-data.asu.edu/)
