# Supplementary material for: Tsunami waves extensively resurfaced the shorelines of an early Martian ocean
Source: Sci Rep. 2016 May 19;6:25106. doi: 10.1038/srep25106 (PMC4872529; doi:10.1038/srep25106)
Supplement: Supplementary Calculations [file srep25106-s2.doc]

**Supplementary calculations of tsunami wave heights from measured run-up distances to: Tsunami waves extensively resurfaced the shorelines of an early Martian ocean**

J. Alexis P. Rodriguez^1,2^, Alberto G. Fairén^3,4^, Kenneth L. Tanaka^5^, Mario Zarroca^6^, Rogelio Linares^6^, Thomas Platz^1,7^, Goro Komatsu^8^, Hideaki Miyamoto^9^, Jeffrey S. Kargel^10^, Jianguo Yan^11^, Virginia Gulick^2,12^, Kana Higuchi^3^, Victor R. Baker^10^, Natalie Glines^2,12^

*^1^Planetary Science Institute, 1700 East Fort Lowell Road, Suite 106, Tucson, AZ 85719-2395, USA.*

*^2^NASA Ames Research Center, Mail Stop 239-20, Moffett Field, CA, 94035, USA.*

*^3^Department of Planetology and Habitability, Centro de Astrobiología (CSIC-INTA), Madrid 28850, Spain.*

*^4^Department of Astronomy, Cornell University, Ithaca, NY 14850, USA.*

*^5^Astrogeology Science Center, U.S. Geological Survey, Flagstaff, AZ 86001, USA.*

*^6^External Geodynamics and Hydrogeology Group, Department of Geology, Autonomous University of Barcelona , 08193 Bellaterra, Barcelona, Spain.*

*^7^Planetary Sciences and Remote Sensing, Institute of Geological Sciences, Freie Universität Berlin, 12249 Berlin, Germany.*

*^8^International Research School of Planetary Sciences, Università d’Annunzio, Viale Pindaro 42, 65127 Pescara, Italy.*

*^9^The University Museum, University of Tokyo, 113-0033, Japan.*

*^10^Department of Hydrology & Water Resources, University of Arizona, Tucson, AZ 85721, USA.*

*^11^State Key Laboratory of Information Engineering in Surveying, Mapping and Remote Sensing, Wuhan University, Wuhan 430070, China.*

*^12^SETI Institute, 189 Bernardo Avenue, Mountain View, CA 94043, USA.*

The most sophisticated numerical model of Martian tsunami waves is that produced by Iijima et al.^1^. Their simulations predict the wave velocities and heights through the ocean’s surface and at the coastline. However, they do not model run-up distances.

A simplified approach that can be utilized to estimate such tsunami run-up distances is the consideration of the momentum balance of a classical long-wave equation with the frictional dissipation for a zero slope surface^2-4^:


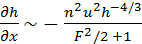
 (1)

The tsunami run-up velocity (*u)* is derived from *F* (*gh*)^1/2^, in which *g* is the gravitational acceleration, *F* is the Froude number, and *h* is the wave’s height.

From the momentum equation it is found^2-4^ that:


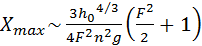
 (2)

where *X_max_* (in m) is the maximum horizontal propagation distance, *h_0_* is the wave’s height at the shoreline, and *n* is the Manning roughness coefficient. Many authors consider that *n* scales inversely with the square root of gravity^5^, implying that, for a given tsunami thickness, *X_max_* might not be significantly influenced by differences in gravitational acceleration.

The Froude number (F ~ 0.5) was firstly approximated considering a wave of a 120 m-height flowing at 10 m/s at the shoreline, which is documented as typical in the numerical simulations by Iijima et al.^1^. For simplicity we assumed that the Froude number to be constant during the run-ups. Under the lower Martian gravity, the waves would have been higher and would have propagated at lower velocities than if they occurred on Earth^1,6^, where higher Froude number value might apply^2^.

The slopes over which the tsunamis propagated are all within small percentages of a degree:

| **Tsunami slopes in degree angles** | | | |
| --- | --- | --- | --- |
| **Profile count** | **Mean** | **Min** | **Max** |
| 71 | 0.074±0.07 | 0.006 | 0.404 |

**Table 1.** showing slopes in degree angles estimated from a total of 71 profiles (Fig. S6).

When these values are included in the calculations of run-up distances, thereby accounting for regional elevation gains, the effect on their horizontal propagation distances is almost negligible:


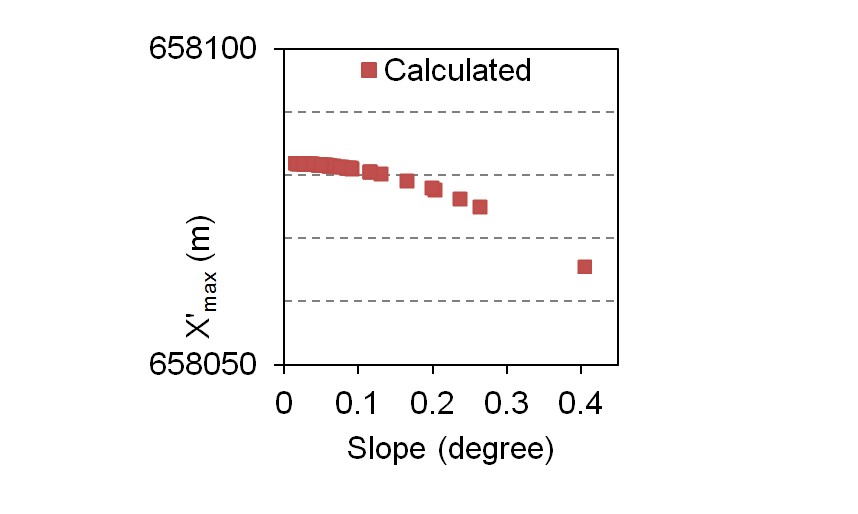


**Plot 1.** showing the run-up distances (X_max_ in equation 2 is modified by a slope coefficient X’_max_ = X_max_·cos (*β*), where (*β*) is the slope angle) solved for a wave 120 m in height using the slope values obtained from the elevation profiles shown in Fig. S6. X_max_ distances calculated from a wave velocity *u* = 10 m/s (F ~ 0.5) and a roughness Manning number *n* = 0.03 (under Mars gravity, considering such gravity effect proportional to ~ g^-1/2^) as input parameters.

Using X_max_ values measured in 71 topographic profiles (Fig. S6), which include both the horizontal and vertical components of the run-up distances, we have calculated the tsunami wave onshore heights. Their values are typically close to ~ 50 m and range between ~ 10 m and ~ 120 m.


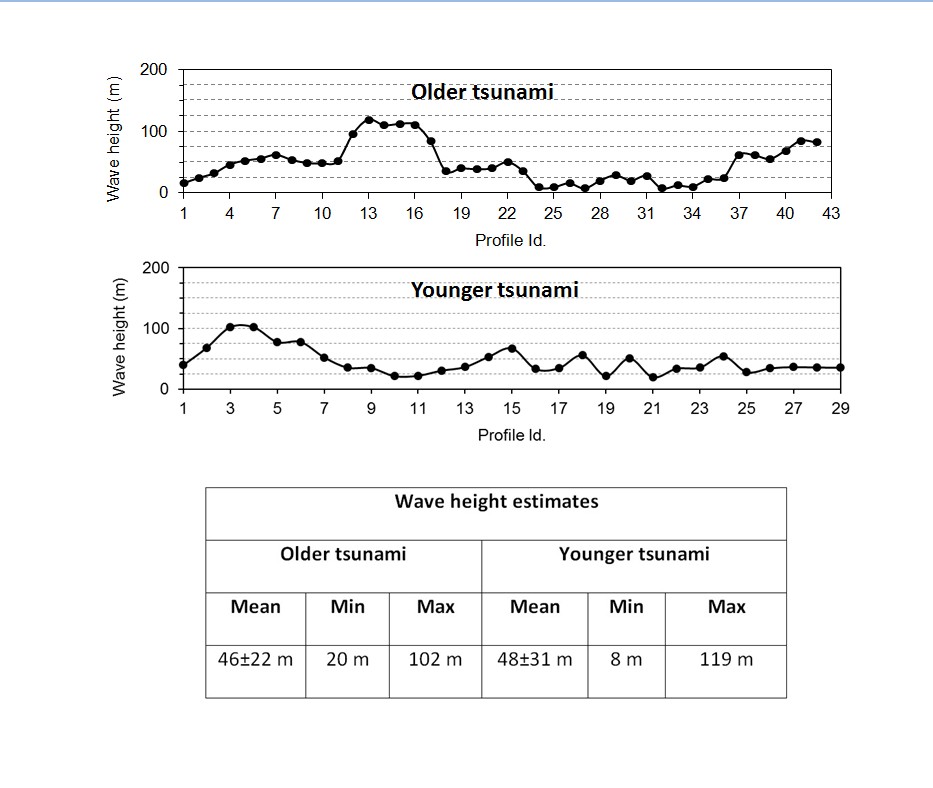


**Plot 2.** showing equation 2 solved for wave elevation heights (*h*_0_) using X_max_ values measured in topographic profiles (Fig. S6) and wave motion input parameters applied in the previous plot.

It should be noted that, because of the large uncertainty in the hydraulics of wave propagation on the Martian surface, these values are considered to be a first approximation.

While this numerical section comprises a first order attempt to fill this gap of knowledge regarding the hydrodynamic character of onshore tsunami propagation, our estimates find close agreement between (1) our estimated run-up distances and the map-based measurements (Plot 1), and (2) our estimated wave heights and those values documented by Iijima et al.^1^, based on their numerical modeling (Plot 2).

**References**

1 Iijima, Y., Goto, K., Minoura, K., Komatsu, G. & Imamura, F. Hydrodynamics of impact-induced tsunami over the Martian ocean. *Planet. Space Sci.* **95**, 33-44 (2014).

2 Bretschneider, C. L. & Wybro P. G. Tsunami inundation prediction. In Proc. of 15th Coastal engineering conference, 1, 1006-1024, Am. Soc. Of Civil Engineering, New York (1977).

3 Hills, J.G. & Mader, C.L., Tsunami produced by the impacts of the small asteroids, *Ann. N.Y. Acad. Sci*. **822**, 381-394 (1997).

4 Pignatelli, C. P. Sanso, & G. Mastronuzzi, Evaluation of tsunami flooding using geomorphologic evidence, *Mar. Geol.* **260**, 6-18 (2009).

5 Komar, P. D. Comparisons of the hydraulics of water flows in Martian outflow channels with flow of similar scale on Earth. *Icarus* **37**, 156- 181 (1979).

6 Mahaney, W. C., Dohm, J., Costa, P., & Krinsley, D. H. Tsunami on Mars: Earth analogues of projected marine sediment. *Planet. Space Sci.* **58**, 1823-1831(2010).
