# Supplementary material for: Tsunami waves extensively resurfaced the shorelines of an early Martian ocean
Source: Sci Rep. 2016 May 19;6:25106. doi: 10.1038/srep25106 (PMC4872529; doi:10.1038/srep25106)
Supplement: Supplementary Crater Statistics [file srep25106-s3.doc]

**Supplementary crater statitistics to: Tsunami waves extensively resurfaced the shorelines of an early Martian ocean**

J. Alexis P. Rodriguez^1,2^, Alberto G. Fairén^3,4^, Kenneth L. Tanaka^5^, Mario Zarroca^6^, Rogelio Linares^6^, Thomas Platz^1,7^, Goro Komatsu^8^, Hideaki Miyamoto^9^, Jeffrey S. Kargel^10^, Jianguo Yan^11^, Virginia Gulick^2,12^, Kana Higuchi^3^, Victor R. Baker^10^, Natalie Glines^2,12^

*^1^Planetary Science Institute, 1700 East Fort Lowell Road, Suite 106, Tucson, AZ 85719-2395, USA.*

*^2^NASA Ames Research Center, Mail Stop 239-20, Moffett Field, CA, 94035, USA.*

*^3^Department of Planetology and Habitability, Centro de Astrobiología (CSIC-INTA), Madrid 28850, Spain.*

*^4^Department of Astronomy, Cornell University, Ithaca, NY 14850, USA.*

*^5^Astrogeology Science Center, U.S. Geological Survey, Flagstaff, AZ 86001, USA.*

*^6^External Geodynamics and Hydrogeology Group, Department of Geology, Autonomous University of Barcelona , 08193 Bellaterra, Barcelona, Spain.*

*^7^Planetary Sciences and Remote Sensing, Institute of Geological Sciences, Freie Universität Berlin, 12249 Berlin, Germany.*

*^8^International Research School of Planetary Sciences, Università d’Annunzio, Viale Pindaro 42, 65127 Pescara, Italy.*

*^9^The University Museum, University of Tokyo, 113-0033, Japan.*

*^10^Department of Hydrology & Water Resources, University of Arizona, Tucson, AZ 85721, USA.*

*^11^State Key Laboratory of Information Engineering in Surveying, Mapping and Remote Sensing, Wuhan University, Wuhan 430070, China.*

*^12^SETI Institute, 189 Bernardo Avenue, Mountain View, CA 94043, USA.*

The counting was performed on Viking MDIM and THEMIS daytime IR data by Werner et al. [1]. Isochrone fits and resultant model ages are based on crater diameters ≥2 km.

Unit lHl1

3.52 Ga (+0.09 Ga, -0.33 Ga), count #29 (central Chryse)

3.48 Ga (+0.06 Ga, -0.12 Ga), count #46 (north-central Chryse)

3.58 Ga (+0.05 Ga, -0.09 Ga), count #36 (Acidalia)

Unit lHl2

3.55 Ga (+0.06 Ga, - 0.12 Ga), count #40 (SW Chryse)

3.57 Ga (+0.10 Ga, -1.00 Ga), count #31 (S Chryse)

3.69 Ga (+0.06 Ga, -0.13 Ga), count #30 (E Tempe)

3.45 Ga (+0.10 Ga, -0.38 Ga), count #35 (Mawrth mouth)

**References**

1 Werner, S.C., Tanaka, K.L., & Skinner, J.A., Jr., 2011, Mars—The evolutionary history of the northern lowlands based on crater counting and geologic mapping. *Plane.Space Sci.* **59**, 1143–1165 (2011).
